# Patient-centered care coordination: a qualitative study of the lived experience of residents in Philadelphia recovery homes

**DOI:** 10.1186/1940-0640-10-S1-A39

**Published:** 2015-02-20

**Authors:** Jennifer Miles, Amy A Mericle, Fred Way, John Cacciola

**Affiliations:** 1 Institute for Behavioral Health, Brandeis University, Waltham, MA, 02454, USA; 2 Alcohol Research Group, Public Health Institute, Emeryville, CA, 94608, USA; 3 National Alliance of Recovery Residences, St. Paul, MN, 55105, USA; 4 Treatment Research Institute, Philadelphia, PA, 19106, USA

## Background

Individuals struggling with a substance use disorder often have a variety of co-occurring service needs that go unmet due to the difficulty in coordinating these services. As the substance use treatment system moves toward treating addiction as a chronic disease, it will be necessary to develop models for the coordination of general medical and addiction treatment services, coupled with a number of social services. Patient-centered care models are one way of improving care coordination by incorporating the goals as expressed by the patient into the treatment plan, which is then coordinated by all members of the treatment team to surround the patient with all necessary resources [1]. Recovery residences, such as recovery homes in Philadelphia, Pennsylvania, could represent a potentially important mechanism for such coordination that is patient centered by providing a stable, supportive, living environment that promotes access to a variety of community-based resources.

## Methods

Using qualitative data from 12 focus groups held with 97 residents in a stratified random sample of Philadelphia recovery homes, the present study provides insight into the potential of these homes to better coordinate care for these individuals and how closely this coordination resembles that of a patient-centered care model. Three independent coders used a manualized coding schema to identify key themes, and consensus was reached when disagreement occurred between codes for any given passage.

## Results

Residents reported having access to medical, substance use, and mental health treatment services, as well as access to community-based services aimed at improving other life domains such as employment and education. Residents also reported that living in these homes provided them with additional social support and assisted in the development of life skills. Although many residents reported having a positive experience, the difficulties of living in a recovery residence were also discussed.

## Conclusions

Qualitative data presented on the lived experiences of these residents highlight the potential of recovery residences to operate in a way that fits closely with a patient-centered care model, and potentially improves access to vital treatment and support services. The need for additional research, as well as policy implications related to the need for standardization of these homes and the potential for inclusion of these residences in the continuum of care for substance use treatment, are discussed.
